# Acceptability and Usability of Mobile Apps for Smoking Cessation Among Young Adults With Psychotic Disorders and Other Serious Mental Illness

**DOI:** 10.3389/fpsyt.2021.656538

**Published:** 2021-05-07

**Authors:** Minda A. Gowarty, Kelly A. Aschbrenner, Mary F. Brunette

**Affiliations:** ^1^Departments of Internal and Family Medicine, Dartmouth-Hitchcock Medical Center, Lebanon, NH, United States; ^2^Geisel School of Medicine at Dartmouth, Hanover, NH, United States; ^3^Center for Technology and Behavioral Health, Geisel School of Medicine at Dartmouth, Lebanon, NH, United States; ^4^Department of Psychiatry, Dartmouth-Hitchcock Medical Center, Lebanon, NH, United States

**Keywords:** smoking cessation, mHealth, serious mental illness, digital health, tobacco treatment, psychiatric illness, schizophrenia, smartphone application

## Abstract

**Background:** Young adults with serious mental illness (SMI) are over twice as likely to smoke cigarettes than those in the general population, but little research has evaluated the efficacy of interventions for this group. While smartphone apps are a promising tool to address this need, their usability should be evaluated among young adults with psychotic disorders, whose symptoms and cognitive impairments may be a barrier to app use.

**Methods:** We compared usability and acceptability of National Cancer Institute apps (QuitGuide and quitSTART) between young adult smokers with SMI psychotic disorders and other SMI diagnoses. We evaluated objective app usability at the initial study visit and following 2 weeks of independent use *via* a video-recorded task-completion protocol. Perceptions of usability and acceptability were assessed with semi-structured interviews. Engagement was assessed with backend app use data.

**Results:** Participants had a mean age of 29 years old (SD = 4). Of the participants without psychotic disorders (*n* = 10), all were diagnosed with SMI post-traumatic stress disorder (SMI-PTSD). QuitGuide objective task completion rates were high and similar between diagnosis groups, whereas quitSTART task completion was initially lower among users with psychotic disorder compared to users with SMI-PTSD at Visit 1, and improved by Visit 2. Mean app interactions, mean days of use, and median completed notifications were dramatically higher among quitSTART users compared to QuitGuide users. Compared to quitSTART users with SMI-PTSD, quitSTART users with psychotic disorders had similar daily app interactions over the first week of use (mean 3.8 ± 2.4 interactions), and numerically lower mean daily app interactions during the second week (1.9 ± 1.5 vs. 3.4 ± 2.5), whereas completed notifications remained stable among quitSTART users in both diagnosis groups over time. Qualitative comments indicated general acceptability of both apps among both diagnosis groups.

**Conclusions:** Both QuitGuide and quitSTART were usable and appealing among young adult smokers with psychotic disorders and SMI-PTSD, although quitSTART engendered a dramatically greater level of engagement compared to QuitGuide. Initial coaching to support initiation and app notifications to promote prolonged engagement may be important for young adult smokers with psychotic disorders. Replication and efficacy testing for quitSTART is warranted.

## Introduction

While the overall rate of smoking in the United States continues to decline, smoking rates remain higher and cessation rates remain lower for people with serious mental illness (SMI; such as schizophrenia and severe mood or anxiety disorders) compared to the general population (1–3). Quitting at a younger age can provide greater health benefits than quitting later (4), making young adulthood an important target for cessation interventions. Significant work has been done on smoking cessation interventions for young adults in the general population (5, 6), and for middle-aged adults with SMI [e.g., (3, 7–9)], however more work is needed to address smoking in young people with SMI. Smoking cessation apps are a promising tool based on testing among general population adults [e.g., (10, 11)], but no research has evaluated their impact in young adults with SMI.

We previously conducted focus groups among young adults with SMI that identified an interest in using smoking cessation apps during a quit attempt as well as an interest in a variety of app features related to smoking and quitting (12). These young adults did not specify an interest in addressing their mental illness within a smoking cessation app, suggesting that apps with evidence-based smoking cessation content designed for the general public may be appealing to this group. However, usability has been identified as a possible problem for people with SMI, particularly those with psychotic disorders such as schizophrenia (13, 14), and therefore warrants additional consideration.

People with psychotic disorders experience a greater degree of cognitive impairments and amotivation than people with other types of SMI (15–18). Such cognitive impairments and amotivation may influence how they interact with digital tools (13, 14). We and others have identified design features that may overcome these barriers (9, 19–21), but it is unknown to what extent general smoking cessation tool designs are problematic for young people with psychotic disorders. Smartphone technology continues to gain popularity across demographic groups (22), and recent data demonstrate that nearly 80% of young adults with SMI own smartphones and most are interested in using their smartphones for health interventions (23). Given that young adults have grown up in an era of smartphone technology and report greater comfort with technology than older age groups (24), it is possible that their familiarity with digital technology may mitigate the effect of cognitive impairments associated with psychotic disorders.

We therefore conducted a study with the primary goal of assessing the usability and acceptability of two smoking cessation smartphone apps designed for general population adults (QuitGuide) and teens (quitSTART) among young adults with SMI (25). The purpose of this secondary analysis was to examine the apps' usability and acceptability among young adult smokers with psychotic disorders compared to smokers with other serious mental illness diagnoses in order to determine whether usability and acceptability differed between these two groups.

## Methods

### Participants and Recruitment

Study methods have been previously reported in detail (25) and will be described briefly here. We recruited young adult smokers who were in treatment for SMI (assessed by the mental health clinic as a serious mental illness of any diagnosis causing substantial disability). Clinicians referred potentially eligible subjects and some self-referred from waiting room flyers. Inclusion criteria included: age 18-35 years old, English-speaking, stable in outpatient mental health treatment for SMI (i.e., stable in treatment with no hospitalization in past 30 days per chart review), self-reported regular tobacco smokers (daily and non-daily) confirmed by breath carbon monoxide (CO) > 7 parts per million (ppm) (26), and smartphone users (either Apple or Android). We selected the relatively broad young adult age range of 18-35 in order to assess the apps in the largest user group that maximizes the potential for the long-term health benefits of smoking cessation, since quitting by age 35 has been associated with life expectancy similar to that of a never smoker (4, 27). Exclusion criteria were current pregnancy or current, unstable substance use disorder per chart review or per the participant's mental health center clinician. Interest in quitting or reducing their smoking was not required to join this study. Prior usability research has demonstrated that over 80% of usability issues can be identified after the first five participants (28), therefore, we aimed to recruit at least five participants with psychotic disorders and five with other SMI diagnoses to use each app (20 total).

### Interventions

QuitGuide and quitSTART are smoking cessation apps available without cost at Smokefree.gov *via* the Apple Store or Google Play. While QuitGuide was designed for adults and quitSTART was designed for teens, the apps share a number of commonalities, including encouraging the user to set a quit date within 14 days, providing informational and motivational content related to quitting smoking, providing users the opportunity to set notifications based on time or location, allowing users to track information such as “slips” (cigarettes smoked) and cravings, and providing feedback to the user based on information they had previously entered into the app.

**Figure 1:**
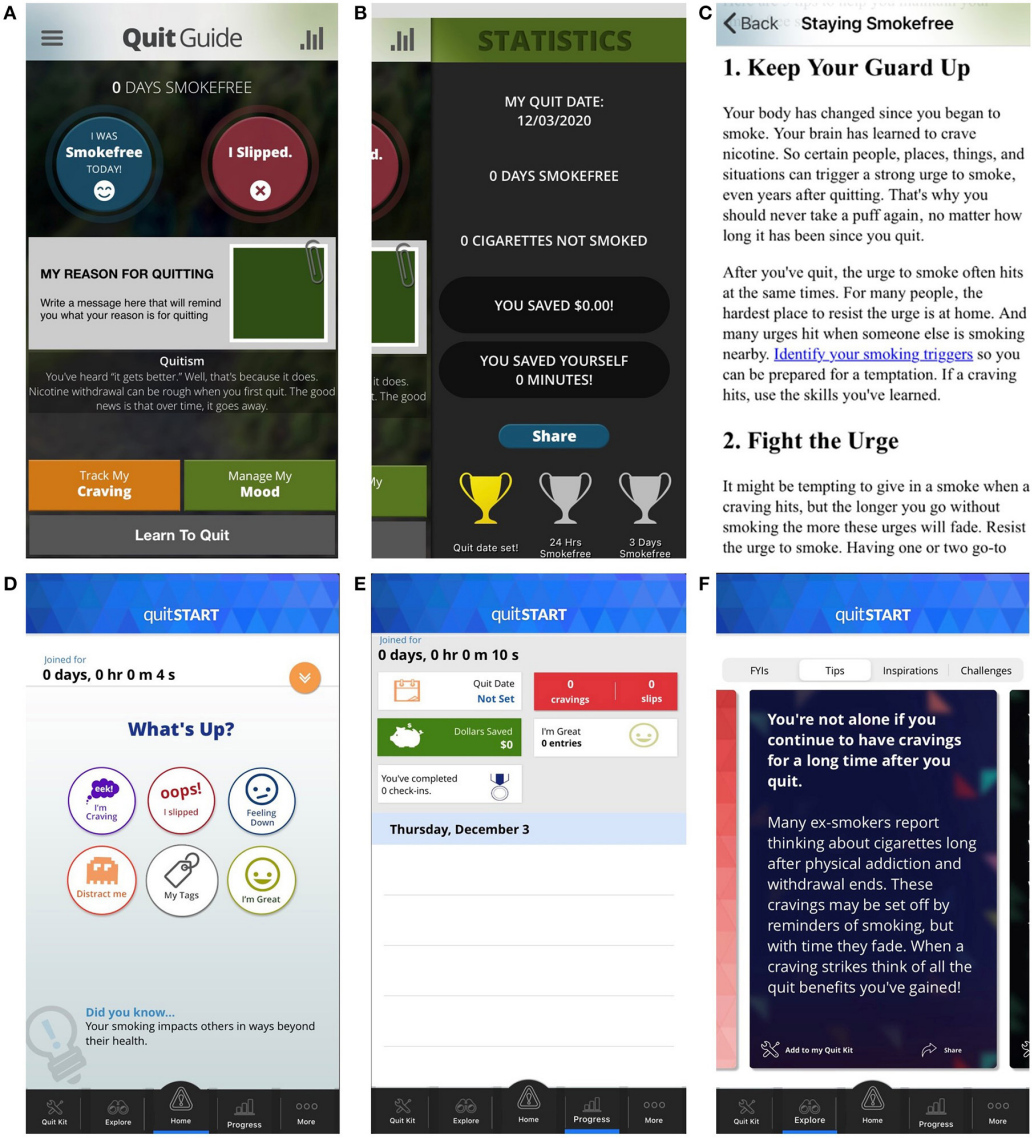
Selected Screen Shots from QuitGuide **(A–C)** and quitSTART **(D–F)**. Represented here are the home screens **(A,D)**, progress screens **(B,E)**, and information on how to quit smoking **(C,F)**.

The two apps differ in several aspects of their content and design (see Table 1 and Figure 1). This includes their layout (linear vs. complex), color palette (dark vs. bright), balance of text and symbols (text-heavy vs. symbol-based), and delivery of information (black and white text document vs. colorful swipeable “cards”). Additionally, quitSTART also automatically sends check-in notifications that ask users how many cigarettes they have smoked since the last check-in, whereas QuitGuide does not have an analogous check-in feature.

**Table 1:** Comparison of QuitGuide and quitSTART characteristics.

	**QuitGuide**	**quitSTART**
Layout	Linear	Complex
Color Palette	Dark	Bright
Balance of Text and Symbols	Text-heavy	Symbol-based
Information Delivery	Black and white text document	Colorful swipeable “cards”
Other Features	Text-only journal	Games

### Procedures

Participation in the study lasted 2 weeks. At the first study visit (Visit 1), participants were randomly assigned to one of the two apps. They completed questionnaires regarding demographics, tobacco use, technology use, and app feature preferences. They then completed the usability protocol described below, followed by a semi-structured interview and usability and acceptability questionnaire items. At the end of Visit 1, participants were instructed to use the app independently for 2 weeks before returning for Visit 2 and repeating the usability protocol, questionnaires, and semi-structured interview.

### Measures

#### Demographics, Technology Use, Diagnosis

Researchers obtained participants' demographics at Visit 1, and history of technology use (e.g., frequency of internet use and app use) at Visits 1 and 2. Researchers reviewed medical record at Visit 1 to obtain Psychiatric diagnosis, mental health stability, and insurance status.

#### Tobacco History and Smoking

With a structured interview at Visit 1, researchers obtained participants' tobacco history (e.g., duration and frequency of smoking, product use, prior quit attempts). At Visits 1 and 2, researchers used the Fagerström test for nicotine dependence (29), a six-item scale shown to be reliable and valid among smokers with SMI (30), to obtain a rating of level of dependence. Smoking status was confirmed with exhaled breath CO > 7 ppm (measured with a Covita Smokerlyzer^®^, Santa Barbara, CA) at both visits (26).

#### Observed App Usability

We developed and administered a usability protocol with basic principles of usability testing (31, 32). Using a “Think Aloud” procedure (33), participants were given up to 5 min to freely explore the app, and then were asked to complete nine specified tasks within the app (Appendix 1). Tasks were chosen based on U.S. Clinical Practice Guidelines (34), as well as prior studies that evaluated frequently used features, desired app features, and features that have been correlated with point prevalence abstinence (12, 35, 36). The participants' phone screens and hand motions were video-recorded as they completed the tasks.

Video recordings were scored as “completed” if the participant was able to reach the requested end point, or “not completed” if the participant requested to skip the task or did not reach the requested end point. “Usability challenges” were defined either as actions performed in the app that could not be used to reach the requested endpoint, or difficulty reaching the requested endpoint identified either by researcher review or by participant verbalization during the task.

#### System Usability Scale

This validated questionnaire (37) is commonly used to assess the usability of many types of technologies, including apps among people with SMI (8, 38, 39). Scores range from 0 to 100, with values of 68-70 representing average usability (40, 41).

#### Perceived Ease of Use and Acceptability Questionnaire

Using questions from the Post-Study System Usability Questionnaire (PSSUQ) (42) and the Usefulness, Satisfaction, and Ease of Use (USE) Questionnaire (43), we administered a 14 item questionnaire, using a five-point Likert-type scale, to assess ease of use and acceptability. We chose a subset of questions from these scales that have been previously used in people with SMI (44). The complete list of questions is presented in Appendix 2.

#### App Perceptions Semi-Structured Interview

In addition to structured questionnaires, we conducted semi-structured qualitative interviews at each visit. We posed open-ended questions to elicit participant feedback on their assigned app's features, content, and ease of use. While we recognized mental illness could affect participants' perceptions of the apps, we elected to use general open-ended questions about the apps rather than specific questions about mental illness or other topics. This allowed participants to discuss what they felt was most important to their experience with their assigned app.

#### App Utilization

We obtained backend app usage data from NCI (45), including date and time of app use, buttons clicked in the app, and response to notifications.

### Participant Flow

Ninety-eight potentially eligible individuals with any SMI diagnosis were screened. Thirty-four were ineligible based on pre-screening criteria, seven refused due to work or child care responsibilities, one due to an upcoming move. Ten were not eligible because they did not have working smartphones, and 27 declined to participate. Researchers obtained informed consent from 19 participants. Of these, two were ultimately deemed ineligible due to low breath CO, resulting in 17 study participants. All 17 participants completed Visits 1 and 2 (100% retention); 15 provided backend app use data (home app use data was not available for two quitSTART participants due to phone problems, and these participants were excluded from the app utilization analyses).

### Data Analysis

#### Quantitative Analyses

All quantitative analyses were completed using descriptive statistics. One usability task was missing at Visit 2 for one participant, thus these data were omitted from the analysis.

For the 15 participants with backend app use data available, we analyzed home app use on days 2-14, thus excluding days with study visits. Complete data was available for all 15 participants on all days except day 14, since one participant was assessed a day early.

#### Qualitative Analyses

Transcriptions of the audio-recorded semi-structured interview responses were compared to the original audio files to ensure accuracy, and then analyzed using thematic analytic techniques (46). Themes were readily apparent due to the descriptive nature of the data, and researchers reached consensus regarding these themes after a single discussion without discrepancies. Negative case analysis was used to ensure the entire data set was represented in the emerging themes.

We reviewed video recordings from the usability task completion protocol to assess participants' ability to reach each task's end point, and to identify usability challenges. Participants' comments during the session were also used to identify usability challenges. During the initial coding of the videos, definitions regarding user challenges were refined until a final set of definitions was reached. Final coding of the video recordings was performed using this set of definitions.

## Results

### Participant Characteristics

Participant characteristics are shown in Table 2. All were daily smokers, 7 (41%) were female, and 16 (94%) were White. Just under half (*n* = 7, 41%) were diagnosed with psychotic disorders (schizophrenia spectrum disorders; hereafter used interchangeably with “psychosis” for readability), and the remainder (*n* = 10, 59%) were diagnosed with SMI post-traumatic stress disorder (SMI-PTSD). Overall, participants smoked an average of 15 cigarettes per day (SD = 7). Most participants (*n* = 15, 88%) had previously engaged in a quit attempt, and all but one (*n* = 16, 94%) used smartphone apps on a daily basis. The psychosis and SMI-PTSD groups were similar, though there was a lower proportion of female participants in the psychosis group (*n* = 1, 14% vs. *n* = 6, 60%).

**Table 2:** Participant Characteristics.

**Characteristic**	**Total**	**Psychosis**	**SMI-PTSD**
	**(*n* = 17)**	**(*n* = 7)**	**(*n* = 10)**
**Demographic and Clinical Characteristics**			
Mean age ± SD	29 ± 3.9	30 ± 3.6	28 ± 4.2
Female, *N* (%)	7 (41)	1 (14)	6 (60)
White, *N* (%)	16 (94)	6 (86)	10 (100)
High school diploma, *N* (%)	14 (82)	5 (71)	9 (90)
Psychotic disorder, *N* (%)	7 (41)	7 (100)	0 (0)
Currently employed (part-time or full-time), *N* (%)	8 (47)	3 (43)	5 (50)
Medicaid/Medicare beneficiary, *N* (%)	16 (94)	7 (100)	9 (90)
**Tobacco Use Characteristics**			
Mean cigarettes/day ± SD	15 ± 7	18 ± 8.5	13 ± 5.3
Mean baseline breath carbon monoxide ± SD	26 ± 11	24 ± 11	27 ± 11
Mean Fagerström Score ± SD	4.4 ± 1.8	5.1 ± 2.1	3.8 ± 1.5
Mean age started smoking ± SD	13 ± 3.5	14 ± 3.0	13 ± 3.9
Prior quit attempt, *N* (%)	15 (88)	7 (100)	8 (80)
**Smartphone Use Characteristics**			
Use smartphone ≥ twice daily, *N* (%)	16 (94)	7 (100)	9 (90)
Use apps at least once per day, *N* (%)	16 (94)	7 (100)	9 (90)
Ever downloaded a health app, *N* (%)	13 (77)	4 (57)	9 (90)

### Usability

**Figure 2:**
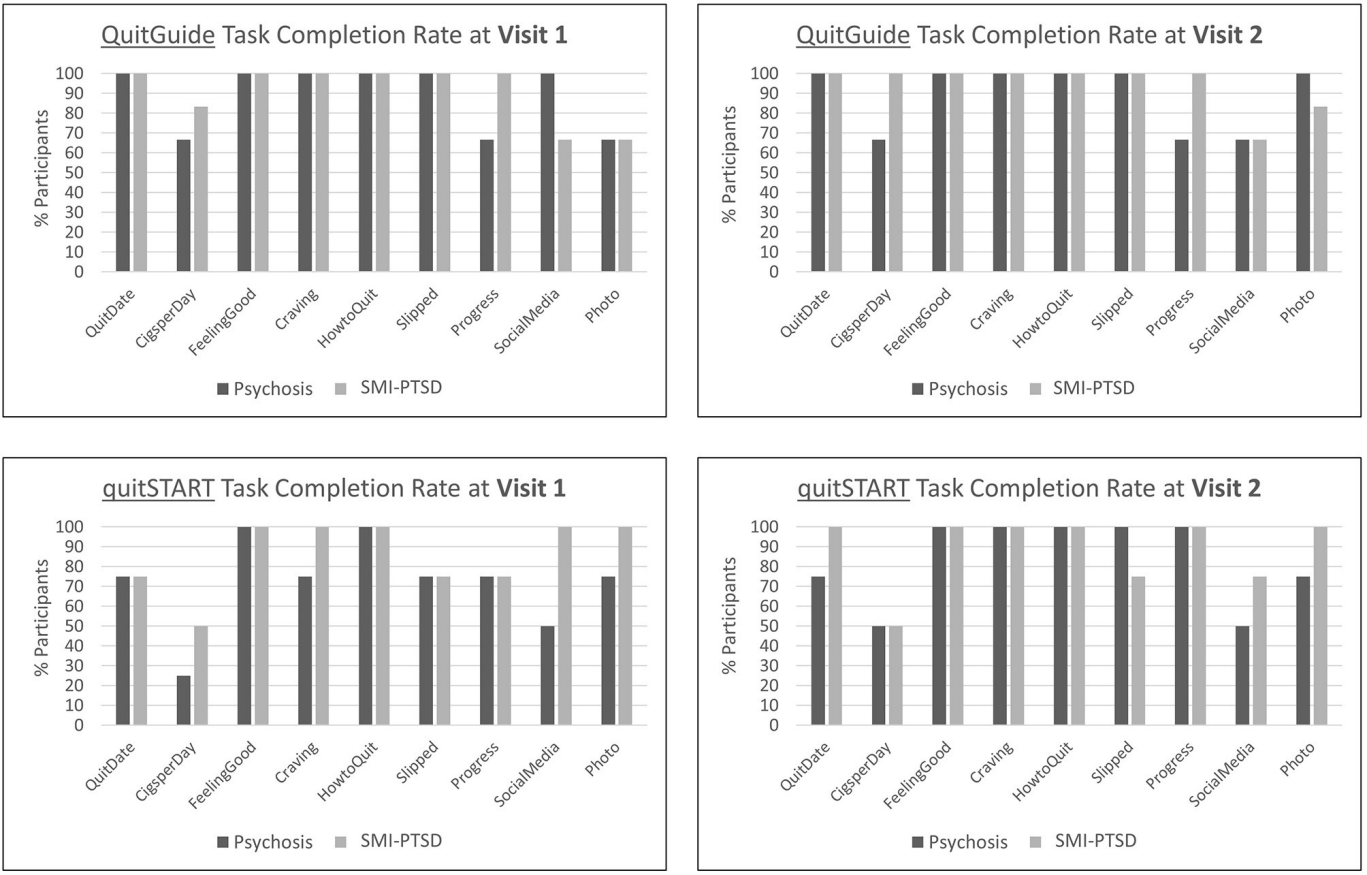
Task completion rates for each app for participants with psychosis and SMI-PTSD.

QuitGuide task completion rates, a key marker of usability, were similar between diagnosis groups, and were high at both at both visits (Figure 2). quitSTART task completion rates were lower at Visit 1 as compared to QuitGuide, particularly for users with psychosis. However, by Visit 2, completion improved for quitSTART users, and rates were similar between diagnosis groups and compared to QuitGuide users.

Self-reported usability (mean SUS scores; not shown) for QuitGuide at both Visit 1 and Visit 2 were higher for participants with psychotic disorders (76 ± 1.4 and 74 ± 7.6, respectively) compared to participants with SMI-PTSD (59 ± 19 and 63 ± 21, respectively). Mean SUS scores for quitSTART at Visit 1 and Visit 2 were similar between groups, and increased after 2 weeks among participants with psychotic disorders (Psychosis: 53 ± 29 and 68 ± 22, respectively; SMI-PTSD: 58 ± 9.6 and 61 ± 6.9, respectively). Responses to other ease of use questions followed this same pattern.

### Acceptability

**Figure 3:**
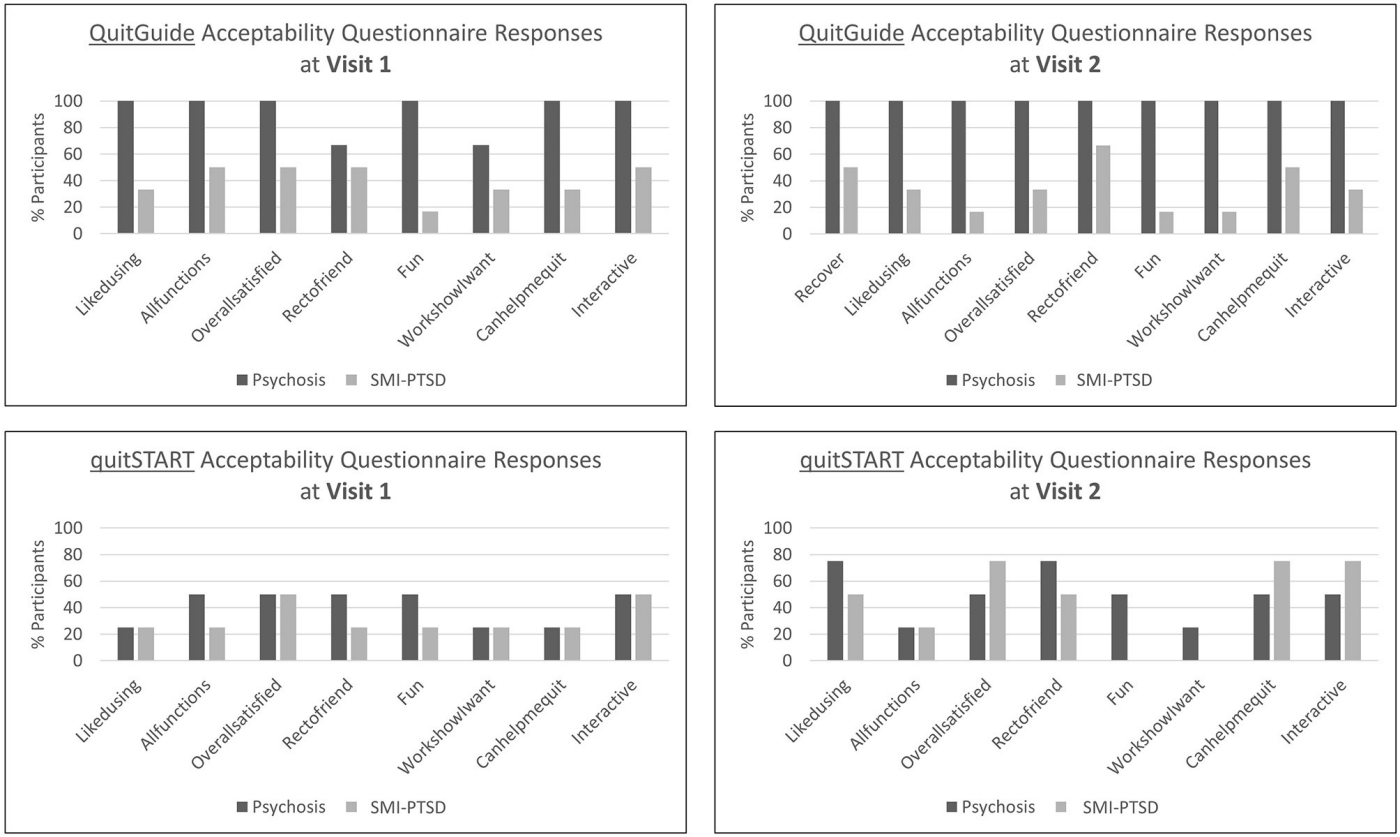
Percent of participants assigned to QuitGuide and quitSTART who agree or strongly agree with the corresponding acceptability statements.

Favorable responses to the structured interview acceptability questions were higher for participants with psychotic disorders than for those with SMI-PTSD for both apps (Figure 3). While responses among QuitGuide users were generally stable from Visit 1 to Visit 2, quitSTART users' responses were low and generally improved at the second visit for both diagnosis categories.

Responses to open-ended questions regarding the apps indicated a higher level of acceptability. A detailed description of participants' semi-structured interview responses is presented elsewhere (25). In summary, participants assigned to both apps expressed positive attitudes toward the apps, though reactions to QuitGuide were generally subdued, while reactions to quitSTART tended to be more exuberant (both positive and negative). Participants perceived that both apps were positive and supportive, and appreciated the motivational content. They noted these aspects of the apps were very important to their ongoing use of the apps. However, both QuitGuide and quitSTART users wished the apps had the ability to track the number of cigarettes smoked per day and felt that the feedback the apps provided to them was inaccurate. While some users did not recall receiving any notifications, and some preferred not to receive notifications, most users assigned to both apps were either satisfied with the number of notifications they received or wished they had received more. The themes of responses to semi-structured interview questions were similar among participants in both diagnosis groups.

### App Utilization

App utilization is a key indicator of both usability and acceptability. Participants interacted with quitSTART dramatically more that QuitGuide over the 13-day study period (Table 3). QuitGuide users in both diagnosis groups opened the app on a similar number of days out of the 2-week period. quitSTART users in both diagnosis groups opened their app on a higher number of days than QuitGuide users, with the greatest days of use among those with SMI-PTSD. Total app interactions followed a similar pattern, with greater interactions among quitSTART users than QuitGuide users, and greater interactions among the SMI-PTSD group.

**Table 3:** Summary of home app use measures over the two-week trial period.

	**QuitGuide**			**quitSTART**		
	**All QuitGuide**	**Psychosis**	**SMI-PTSD**	**All quitSTART**	**Psychosis**	**SMI- PTSD**
	**(*n* = 9)**	**(*n* = 3)**	**(*n* = 6)**	**(*n* = 6)**	**(*n* = 3)**	**(*n* = 3)**
Mean App Interactions ± SD	5.6 ± 3.8	4.7 ± 1.5	6.0 ± 4.7	41 ± 26	33 ± 23	49 ± 32
Mean Days of Use ± SD	4.6 ± 2.8	4.0 ± 2.0	4.8 ± 3.3	11 ± 3.5	9.3 ± 4.7	12 ± 1.2
Median Notifications Completed (Range)	1 (0-8)	1 (0-5)	0.5 (0-8)	19 (0-37)	14 (0-31)	23 (13-37)

**Figure 4:**
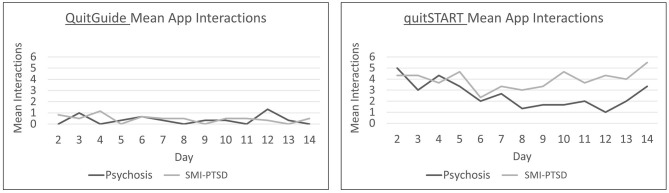
Mean daily app interactions for participants with psychosis and SMI-PTSD.

Mean daily app interactions are shown in Figure 4. QuitGuide users interacted with their app dramatically less than quitSTART users [less than once vs. 3.6 (SD = 2.4) times per day on average during the first week of the trial]. quitSTART app interactions declined among participants with psychosis during the second week compared to participants with SMI-PTSD (1.9 ± 1.5 vs. 3.9 ± 2.4).

**Figure 5:**
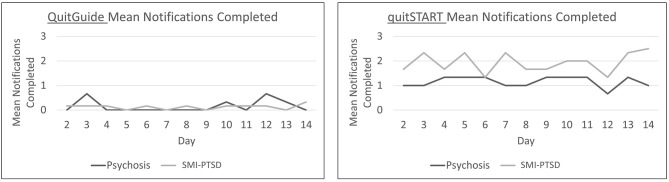
Mean daily completed notifications for participants with psychosis and SMI-PTSD.

Responses to notifications are shown in Figure 5. QuitGuide users in both groups completed very few notifications over the trial period. In contrast, quitSTART users steadily completed more notifications than QuitGuide users overall, with slightly higher completion rates for participants with SMI-PTSD (psychosis 1.2 ± 1.2 vs. SMI-PTSD 1.9 ± 0.9).

### Tobacco Use at Follow-Up

While participants were not required to quit or reduce their smoking for this study, over three-quarters of participants (13/17, 77%) reported attempting to do so during the 2-week trial period. This included 6/7 (86%) of participants with psychosis and 7/10 (70%) of participants with SMI-PTSD. The proportion of participants who reported attempting to quit or reduce their smoking was similar for each app: 7/9 (78%) QuitGuide users, and 6/8 (75%) quitSTART users. Notably, two quitSTART participants reported that they were no longer smoking at Visit 2 (confirmed with breath CO < 7 ppm) after switching completely to e-cigarettes (one with psychosis and one with SMI-PTSD). Both stated that they believed the app had helped them quit smoking.

## Discussion

### Main Findings

In this secondary analysis of usability and acceptability of two smoking cessation apps designed for the general population, young adults with and without psychotic disorders had high objective task completion rates for both apps. Participants with psychotic disorders rated both apps more favorably than those with SMI-PTSD, yet back end app use data demonstrated low engagement with QuitGuide and high engagement with quitSTART among both groups. Participants with psychosis demonstrated declining interactions with quitSTART during their second week of use, while responses to notifications were stable during this time, suggesting that the decline in app interactions was due to a decline in spontaneous app use. Most participants reported attempting to cut down or quit, and two quitSTART participants, one in each diagnosis group, demonstrated biologically verified abstinence from smoking after 2 weeks of app use. Notably, while questionnaire assessment of acceptability suggested low acceptability of the apps, interview responses, user engagement with the apps, quit attempts, and abstinence demonstrated favorable acceptability, especially for quitSTART, among people with psychotic disorders and with SMI-PTSD.

These results suggest several important points regarding app use for smoking cessation among young adults with psychotic disorders, whose mental-illness related cognitive impairments can reduce functional capacity beyond that seen in other SMI diagnoses. First, this form of intervention is very appealing for both young adults with psychotic disorders and young adults with SMI-PTSD. The high level of engagement aligns with our previous survey in which 75% of young adults with SMI were interested in using the devices for health interventions (23). Second, young adults with psychotic disorders were able to easily learn how to use these apps despite several design problems we have previously described (25), indicating that high quality “off the shelf” apps may have utility in this population. Third, young adults with psychotic disorders were activated by the content of the quitSTART app in a manner similar to smokers with SMI-PTSD, suggesting that tailoring content specific for psychotic disorders in this age group may not be required. Finally, the pattern of quitSTART use in those with psychotic disorders (declining spontaneous use while responses to notifications persisted) suggests that using push notifications may be a key strategy to maintain engagement among users with psychotic disorders that warrants testing in future research.

### Comparison With Prior Research

One other small study has assessed usability of QuitGuide, in which QuitGuide was compared to an Acceptance and Commitment Therapy (ACT)-based app developed for people with SMI (Learn To Quit) among middle-aged adults (39). This study included four participants with psychotic disorders who used QuitGuide during the trial, with an average SUS score for QuitGuide of 61.9 (range 35-82.5). While not reported by the presence or absence of psychotic disorder diagnosis, the authors found that in general, participants used QuitGuide self-tracking features more frequently than they responded to Learn to Quit's ecological momentary assessment (EMA) features, leading the authors to hypothesize that notifications may not improve user engagement. These results contrast with our findings among young adults with SMI, in which QuitGuide engagement remained very low over time, and quitSTART users engaged extensively and steadily with notifications. Our findings would suggest that notifications can effectively engage users, particularly among people with psychotic disorders.

Previous evaluations of the usability and acceptability of apps in middle-aged adults with SMI (20, 47) demonstrated that participants found “text-heavy” apps (such as QuitGuide) to be unappealing and difficult to understand, aligning with our finding of low spontaneous use of QuitGuide over the study period. Our participants' report of the importance of a positive, motivational tone also overlaps with findings among middle-aged adults with SMI (9). However, our young adult participants did not mention the need for an app specifically tailored to address mental illness. Whether, digital interventions for smoking among people with SMI require specific mental health content is a topic needing further study.

A secondary analysis of app engagement among middle-aged adults in the general population found that a number of participant characteristics predicted lower app engagement, including lower education level (high school or less), heavier smoking (>10 cigarettes per day), and greater depressive symptoms (48). While our study did not directly assess the impact of these factors on engagement, people with psychotic disorders tend to have lower educational attainment and higher levels of nicotine dependence than those without psychotic disorders, and may be at risk for lower levels of engagement with cessation apps. Thus, our finding that young adults with psychotic disorders exhibited strong engagement with quitSTART further supports quitSTART's potential role in this population.

## Limitations

This usability study was small by design, thus the finding of differences between participants with and without psychotic disorder diagnoses require confirmation in larger studies. Additionally, the 2-week study period was relatively short. Given that smoking and app use patterns change over time, future studies should include longer follow-up. We also did not ask participants about how their mental illness affected their perceptions and use of the apps, nor were we able to assess cognitive abilities and symptom severity in order to examine the relationship between these symptoms and usability. Nevertheless, these novel findings generally align with previous work, which supports their validity.

## Conclusions

Both QuitGuide and quitSTART were usable and acceptable among young adult smokers with psychotic disorders and SMI-PTSD. quitSTART engendered a greater level of engagement compared to QuitGuide, although spontaneous engagement declined while notification engagement persisted among smokers with psychotic disorders. Initial coaching to support initiation and app notifications to promote ongoing engagement may be advantageous for use of quitSTART among young adult smokers with psychotic disorders. Replication in larger study samples and testing for efficacy of quitSTART are warranted.

## Data Availability Statement

The raw data supporting the conclusions of this article will be made available by the authors, without undue reservation.

## Ethics Statement

The studies involving human participants were reviewed and approved by New Hampshire Department of Health and Human Services Institutional Review Board. The patients/participants provided their written informed consent to participate in this study.

## Author Contributions

MG and MB designed the study and prepared the original draft of the manuscript. MG collected the data and performed quantitative and qualitative data analysis with oversight of these activities provided by MB. KA provided expert consultation for data interpretation and reviewed and provided comments on the manuscript before submission. All authors contributed to the article and approved the submitted version.

## Conflict of Interest

The authors declare that the research was conducted in the absence of any commercial or financial relationships that could be construed as a potential conflict of interest.
