# Supplementary material for: Acceptability and Usability of Mobile Apps for Smoking Cessation Among Young Adults With Psychotic Disorders and Other Serious Mental Illness
Source: Front Psychiatry. 2021 May 7;12:656538. doi: 10.3389/fpsyt.2021.656538 (PMC8138181; doi:10.3389/fpsyt.2021.656538)
Supplement: Supplementary file 1 [file Data_Sheet_1.DOCX]

Supplementary Material

# Appendix 1. Usability Tasks

1. Set a new quit date.

2. Tell the app how many cigarettes per day you are smoking.

3. Tell the app that you’re feeling good right now.

4. Tell the app that you want a cigarette right now.

5. Find information on things you can do to help you quit or reduce your smoking. Please read a little bit of what you find out loud.

6. Tell the app that you smoked a cigarette today.

7. Find where there is information on how much you have been smoking (or how many cigarettes you’ve avoided) since you downloaded the app.

8. Find out how you can connect with other people on social media about quitting or reducing smoking.

9. Find where in the app you can take a photo or upload a photo.

# Appendix 2. Ease of Use and Acceptability Questionnaire

1. Overall, I am satisfied with how easy it is to use the app.

2. I felt comfortable using the app.

3. It was easy to learn to use the app.

4. Whenever I made a mistake using the app, I could recover quickly and easily.

5. It was easy to find the information I needed.

6. How things appeared on the screen was clear.

7. I liked using the app.

8. The app has all the functions and capabilities I expect it to have.

9. Overall, I am satisfied with the app.

10. I would recommend the app to a friend.

11. The app is fun to use.

12. The app works the way I want it to.

13. The app can help me quit smoking.

14. The app was interactive enough.
